# Prolyl hydroxylase domain 2 deficiency promotes skeletal muscle fiber-type transition via a calcineurin/NFATc1-dependent pathway

**DOI:** 10.1186/s13395-016-0079-5

**Published:** 2016-03-05

**Authors:** Junchul Shin, Aki Nunomiya, Yasuo Kitajima, Takashi Dan, Toshio Miyata, Ryoichi Nagatomi

**Affiliations:** 1 Department of Medicine & Science in Sport & Exercise, Tohoku University School of Medicine, 2-1 Seiryo-machi, Aoba-ku, Sendai, Miyagi 980-8575 Japan; 2 Division of Biomedical Engineering for Health and Welfare, Tohoku University Graduate School of Biomedical Engineering, 2-1 Seiryo-machi, Aoba-ku, Sendai, Miyagi 980-8575 Japan; 3 Division of Molecular Medicine and Therapy, United Centers for Advanced Research and Translational Medicine (ART), Tohoku University School of Medicine, 2-1 Seiryo-machi, Aoba-ku, Sendai, Miyagi 980-8575 Japan; 4 Center for Sports Medicine and Science, United Centers for Advanced Research and Translational Medicine (ART), Tohoku University School of Medicine, 2-1 Seiryo-machi, Aoba-ku, Sendai, Miyagi 980-8575 Japan

**Keywords:** Hypoxia-inducible factor α, Prolyl hydroxylate domain protein 2, Type I muscle fiber, Calcineurin, NFATc1

## Abstract

**Background:**

Hypoxia exposure is known to induce an alteration in skeletal muscle fiber-type distribution mediated by hypoxia-inducible factor (HIF)-α. The downstream pathway of HIF-α leading to fiber-type shift, however, has not been elucidated. The calcineurin pathway is one of the pathways responsible for slow muscle fiber transition. Because calcineurin pathway is activated by vascular endothelial growth factor (VEGF), one of the factors induced by HIF-1α, we hypothesized that the stabilization of HIF-1α may lead to slow muscle fiber transition via the activation of calcineurin pathway in skeletal muscles. To induce HIF-1α stabilization, we used a loss of function strategy to abrogate Prolyl hydroxylase domain protein (PHD) 2 responsible for HIF-1α hydroxylation making HIF-1α susceptible to ubiquitin dependent degradation by proteasome. The purpose of this study was therefore to examine the effect of HIF-1α stabilization in PHD2 conditional knockout mouse on skeletal muscle fiber-type transition and to elucidate the involvement of calcineurin pathway on muscle fiber-type transition.

**Results:**

PHD2 deficiency resulted in an increased capillary density in skeletal muscles due to the induction of vascular endothelial growth factor. It also elicited an alteration of skeletal muscle phenotype toward the type I fibers in both of the soleus (35.8 % in the control mice vs. 46.7 % in the PHD2-deficient mice, *p* < 0.01) and the gastrocnemius muscle (0.94 vs. 1.89 %, *p* < 0.01), and the increased proportion of type I fibers appeared to correspond to the area of increased capillary density. In addition, calcineurin and nuclear factor of activated T cell (NFATc1) protein levels were increased in both the gastrocnemius and soleus muscles, suggesting that the calcineurin/NFATc1 pathway was responsible for the type I fiber transition regardless of PGC-1α, which responded minimally to PHD2 deficiency. Indeed, we found that tacrolimus (FK-506), a calcineurin inhibitor, successfully suppressed slow fiber-type formation in PHD2-deficient mice.

**Conclusions:**

Taken together, stabilized HIF-1α induced by PHD2 conditional knockout resulted in the transition of muscle fibers toward a slow fiber type via a calcineurin/NFATc1 signaling pathway. PHD2 conditional knockout mice may serve as a model for chronic HIF-1α stabilization as in mice exposed to low oxygen concentration.

**Electronic supplementary material:**

The online version of this article (doi:10.1186/s13395-016-0079-5) contains supplementary material, which is available to authorized users.

## Background

Mammalian skeletal muscle consists of four muscle fiber isoforms: myosin heavy chain type I (slow) and three subtypes of type II (fast) fibers (*a*, *b*, and *x*) [1]. Skeletal muscle fiber-type composition is known as one of the essential determinants of muscle contraction capacity [2]. Although skeletal muscle fiber type is mainly determined by genetic factors, it is regulated by environmental factors such as exercise training [3] or regenerative processes after muscle damage [4]. Specifically, calcineurin/nuclear factor activated T cells (NFAT) signaling and peroxisome proliferator-activated receptor γ coactivator-1α (PGC-1α) are strongly linked to oxidative muscle fiber-type transition in skeletal muscle [5, 6].

Hypoxia causes biological changes that are mediated by the hypoxia-inducible factor-α (HIF-α), a key transcription factor for various compensatory responses under hypoxia [7]. HIF-α is implicated in muscle cell development, vascular formation, and energy metabolism in skeletal muscle [8–11]. Interestingly, recent reports suggested that the exposure to 8 % hypoxic condition showed the shift of the soleus muscle fiber type toward type I phenotype in mice [12]. In another previous study, it was demonstrated that HIF-1α knockdown suppressed the increase of myosin heavy chain (MyHC) I messenger RNA (mRNA) in cultured C2C12 myotubes compared with those cultured under 4 % oxygen in vitro [13]. Although these studies imply the stabilization of HIF-1α is strongly associated with the expression of slow myosin heavy chain, the downstream pathway leading to fiber-type shift to type I has not been elucidated.

Under normoxic conditions, HIF-α subunits, including HIF-1α and HIF-2α, are rapidly hydroxylated by prolyl hydroxylase domain proteins (PHDs), which result in the ubiquitination and proteasomal degradation of HIF-α isoforms [14, 15]. Under hypoxic conditions, however, hypoxia-inducible factor (HIF) is stabilized by the inactivation of PHDs [16, 17]. The three PHD isoforms, PHD1, 2, and 3, are involved in a variety of tissues in response to hypoxic conditions [18, 19]. PHD2 but not PHD1 or 3 is the main isoform responsible for controlling HIF levels under normoxia [20] and is a key factor in angiogenesis and erythropoiesis through the stabilization of HIF-1α and HIF-2α, because HIF-α induces vascular endothelial growth factor (VEGF) and erythropoietin (EPO) production which support angiogenesis and erythropoiesis [21–23]. Interestingly, VEGF is reportedly associated with calcineurin/NFAT signaling in endothelial cell [24]. Since the calcineurin/NFATc1 signaling pathway supports oxidative or slow-type skeletal muscle fiber transition, we hypothesized that HIF-α stabilization may result in the alteration of skeletal muscle fiber type toward the oxidative phenotype via the calcineurin/NFATc1 signaling pathway.

To induce HIF-1α stabilization, we used a loss of function strategy to abrogate PHD2 responsible for HIF-1α hydroxylation making HIF-1α susceptible to ubiquitin dependent degradation by proteasome. The purpose of this study was therefore to examine the effect of HIF-1α stabilization in PHD2 conditional knockout mouse on skeletal muscle fiber-type transition and to elucidate the involvement of calcineurin pathway on muscle fiber-type transition under HIF-1α stabilization.

## Methods

### Ethical approval

All animal experimental procedures were performed according to the protocols approved by the Guidelines for the Care of Laboratory Animals of Tohoku University Graduate School of Medicine (Sendai, Japan).

### Animals

The PHD2^f/+^ mice were generated previously (*f* denotes floxed allele). To delete floxed *Phd2* exon 2, tamoxifen (Sigma, St Louis, Mo) was administered to 12- to 14-week-old *Phd2* ^*f/f*^ */Rosa26* ^*CreERT2*^ mice by forced gavages (10 mg/ml in corn oil, 20 mg/kg/day for seven consecutive days). The Rosa26^CreERT2^ mice used to establish the *Phd2* ^*f/f*^ */Rosa26* ^*CreERT2*^ colony were provided by Connecticut University. To characterize skeletal muscle phenotype, soleus and gastrocnemius were harvested from *Phd2* ^*f/f*^ and *Phd2* conditional knockout (cKO) mice after the mice were sacrificed by cervical dislocation, and then the muscle samples were stored at −80 °C. FK-506 (Enzo Life Sciences) was dissolved in ethanol and phosphate-buffered saline (PBS), and it was administered subcutaneously; the dose of FK-506 was 1 mg/kg/day for seven consecutive days. To monitor the deletion of PHD2 exon 2, as well as the presence of the Rosa 26CreERT2 allele, tail DNA samples were prepared and used for polymerase chain reaction (PCR) before and after tamoxifen administration. A hypoxic chamber, in which the oxygen concentration was regulated by an oxygen controller (ProOx; BioSpherix) with a nitrogen generator (Nilox; Sanyo Electronic Industries), was used to expose the mice to hypoxic condition. For acclimation to severe hypoxia (10 % 02), the mice were exposed to 12 % oxygen for 24 h before the oxygen concentration was reduced to 10 %.

### Cell culture

Mouse C2C12 myoblasts were cultured under standard conditions (37 °C under a humidified atmosphere containing 5 % CO_2_) in high-gulcose Dulbecco’s modified Eagle’s medium (DMEM) supplemented with 10 % fetal bovine serum (Hyclone Laboratories, Logan, UT) and 100 μg/ml penicillin-streptomycin solution. C2C12 cells were differentiated into myotubes by culturing medium (DMEM containing 2 % horse serum, Gibco), when they reached 70–80 % confluence. The cells were transfected with 50 nM small interfering RNA (siRNA) using Lipofectamine RNAimax (Invitrogen). siRNA (Stealth RNAi, Invitrogen) and negative control siRNA (Invitrogen) were used. The medium was then replaced daily for 3 days before analysis [25]. C2C12 cells were harvested in the lysate buffer (40 mM Tris (pH 7.5), 300 mM KCl, 1 % Triton X-100, 0.5 M EDTA, Protease inhibitor cocktail X 20 (Sigma)) using a cell scraper at 72 h after the transfection.

### Blood analysis

Blood samples (0.5–1.0 ml) were obtained from mouse facial veins. Blood profiles (red blood cells, hematocrit, and hemoglobin) were measured using a multi-automatic blood cell counter for animals (MICRO abc LC-152, Horiba, Tokyo, Japan).

### Western blot analysis

To isolate total protein extracts, 50 mg of skeletal muscle tissue was homogenized for 30 s on ice in 1 ml of lysate buffer (40 mM Tris (pH 7.5), 300 mM KCl, 1 % Triton X-100, 0.5 M EDTA, Protease inhibitor cocktail X 20 (Sigma)), using a Polytron PT-MR 2100 homogenizer. Homogenates were centrifuged at 12,000 rpm for 5 min at 4 °C, and the supernatants were isolated. Protein concentrations were determined using the BCA protein assay kit (Thermo Fisher Scientific, Rockford, IL) with bovine serum albumin (BSA) as the standard, and extracts were stored at −80 °C. Nuclear and cytoplasmic extractions were performed using the NE-PER nuclear and cytoplasm extraction reagents (78833, Thermo Fisher Scientific).

Total protein was separated via 8–12 % SDS-PAGE and transferred to a PDVF membrane (Invitrogen). The membrane was blocked using Tris-buffered saline with 0.05 % Tween 20 (TBST) containing 5 % BSA for 1 h and incubated overnight with appropriately diluted (1:500–1000) primary antibody in TBST at 4 °C. After incubation, membranes were rinsed three times in TBST for 5 min and incubated with secondary antibody in 4 % skim milk for 1 h at room temperature. Protein bands were visualized and quantified using a Molecular Imager VersaDoc 5000MP system (Bio-Rad) and ECL.

### Antibodies

The following primary antibodies were used: PHD-2/Egln1 antibody (3293, Cell Signaling Technology), EGLN3/PHD3 antibody (NB100-303, Novus), Monoclonal Anti-Myosin slow (M8421, Sigma), MYH2 antibody (A4.74, Santa Cruz Biotechnology), Anti-PGC-1α (AB3243, Millipore), Anti-Calcineurin pan A (07-1491, Millipore), HIF-1α (NB100-479, Novus), NFATc1 (sc-7294, Santa Cruz Biotechnology), MEF2 (sc-313, Santa Cruz Biotechnology), myoglobin (FL-154, Santa Cruz Biotechnology), β-actin (AC-74, Sigma-Aldrich), GAPDH (14C10, Cell Signaling Technology), and α-tubulin (DM1A, Cell Signaling Technology).

### Determination of muscle fiber-type composition and NFATc1 nuclear translocation

Gastrocnemius and Soleus muscle samples were harvested from PHD2^f/f^ and PHD2 cKO mice (five numbers of animals per group), snap frozen, and embedded in O.C.T. compound (4583, Sakura) in methylbutane for cryosectioning. Skeletal muscle cryosections were air-dried for 10 min and fixed with 4 % paraformaldehyde (PFA) for 15 min. For muscle fiber-type composition experiments, sections were blocked in PBS containing 0.3 % Triton X-100 and M.O.M. blocking reagent (Vector Laboratories) for 1 h at room temperature. Primary antibodies were incubated overnight at 4 °C. We used several primary antibodies, such as Monoclonal Anti-Myosin slow (M8421, Sigma), myosin heavy chain 2A (SC-71, Developmental Studies Hybridoma Bank, Iowa), anti-laminin antibody (L9393, Sigma-Aldrich), and NFATc1 antibody (sc-7294, Santa Cruz Biotechnology) in the blocking solution with M.O.M. protein concentrate (Vector Laboratories). Section samples were incubated with secondary antibodies in PBS for 1 h at room temperature. We used several secondary antibodies, such as Alexa Fluor 488 Goat Anti-mouse IgG and Alexa Fluor 555 Goat Anti-rabbit IgG in PBS containing 0.3 % Triton X-100. All slides were covered with Vectashield mounting medium with DAPI (Vector Laboratories). Samples were visualized on a microscope (C2+, Nikon) and analyzed using NIS elements and ImageJ software. For fiber-type analysis, all fibers within the entire muscle/cross section were characterized. The proportion of muscle fiber type was determined as counting stained fibers by anti-slow myosin and anti-myosin heavy chain 2A.

### Determination of capillary density

Frozen sections were blocked in PBS containing 0.3 % Triton X-100 and 10 % goat serum (Sigma) for 1 h at room temperature. Anti-mouse CD31 (PECAM-1, BD Pharmingen) was used as the primary antibody and was incubated overnight in blocking solution at 4 °C. Alexa Fluor 488 Goat Anti-Rat IgG in blocking solution was used as the secondary antibody for 1 h at room temperature. All slides were covered with Vectashield mounting medium (Vector Laboratories). Detection was performed on a microscope (C2+, Nikon), and images were analyzed using NIS elements software. The fields of whole muscle tissue cross section from soleus and gastrocnemius were entirely selected for CD31-positive cells counting and analyzed using ImageJ software.

### RNA extraction and real-time quantitative PCR

Total RNA from muscle tissues was isolated using the RNeasy Mini Kit (Qiagen) according to the manufacturer’s protocol. Complementary DNA (cDNA) templates were obtained via reverse transcription of 2 μg of total RNA (QuantiTect Reverse Transcription; Qiagen). Quantitative reverse transcription polymerase chain reaction (qRT-PCR) was performed in a 96-well plate format using the Fast SYBR Green PCR master mix and the StepOnePlus Real-Time PCR system (Applied Biosystems). The thermal cycling conditions were as follows: 95 °C for 7 min, followed by 40 cycles of 95 °C for 10 s and 60 °C for 30 s, and a final extension of 95 °C for 15 s and 60 °C for 30 s. Relative expression was calculated using the standard method. All values for each gene were averaged and normalized to β-actin or 18S as an internal control. The following primer sequences were used: *Phd2* forward, 5′-GCCCAGTTTGCTGACATTGAAC-3′, *Phd2* reverse, 5′-CCCTCACACCTTTCTCACCTGTTAG-3′; *Vegf-A* forward, 5′CCACGTCAGAGAGCAACATCA-3′, *Vegf-A* reverse 5′-TCATTCTCTCTATGTGCTGGCTTT-3′; *β-actin* forward, 5′-CTGGGTATGGAATCCTGTGG-3′, *β-actin* reverse, 5′-GTACTTGCGCTCAGGAGGAG-3′; *18S* rRNA forward, 5′-TGGTTCCTTTGGTCGCTCGCTCCTC-3′, *18S* rRNA reverse 5′-TTCCTTGGATGTGGTAGCCGTTTCTCA-3′; *Hif-1α* forward, 5′-CAAGATCTCGGCGAAGCAA-3′, *Hif-1α* reverse 5′-GGTGAGCCTCATAACAGAAGCTTT-3′; *Phd1* forward, 5′-GGTACGTGAGGCATGTTGACAATC-3′, *Phd1* reverse, 5′-TCAAAGAGTGGCTCGATGTTGG-3′; *Phd3* forward, 5′-TCAACTTCCTCCTGTCCCTCATC-3′, *Phd3* reverse, 5′-GCGAACATAACCTGTCCCATTTC-3′; *FBXO32(Atrogin-1)* forward, 5′-ATGCACACTGGTGCAGAGAG-3′, *FBXO32(Atrogin-1)* reverse, 5′-TGTAAGCACACAGGCAGGTC-3′; *Murf-1* forward, 5′-ACCTGCTGGTGGAAAACATC-3′, *Murf-1* reverse, 5′-CTTCGTGTTCCTTGCACATC-3′; and *Sirt-1* forward, 5′-GGCTACCGAGACAACCTCCTG-3′, *Sirt-1* reverse, 5′-AGTCCAGTCACTAGAGCTGGC-3′.

### Immunoassay

VEGF concentrations were determined from gastrocnemius samples from *Phd2* ^*f/f*^ and *Phd2* cKO mice using a mouse VEGF Immunoassay kit (R&D system, Japan). Briefly, muscle samples were prepared from gastrocnemius homogenates in 1.5 ml of ×1 PBS. The homogenates were subjected to two freeze-thaw cycles to break the cell membranes and centrifuged for 5 min at 5000×*g*. The VEGF immunoassay was carried out according to the manufacturer’s protocol.

### Statistics

All results are expressed as the means, with error bars representing the standard error. Two-tailed Student’s *t* tests and one-way ANOVA were performed to determine *p* values. Statistical significance was defined as *p* < 0.05. Analyses were performed using IBM SPSS Statistics 19.0.

## Results

### PHD2 expression is efficiently reduced by tamoxifen administration

**Fig. 1:**
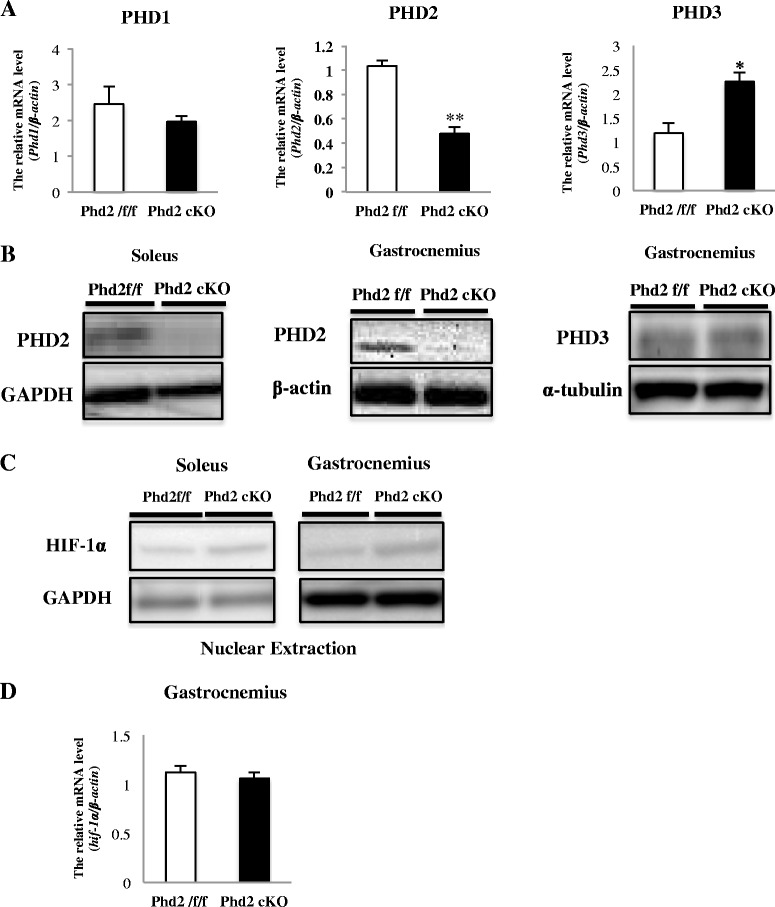
Tamoxifen administration-induced PHD2 deletion in skeletal muscle in *Phd2* ^*f/f*^ */Rosa26* ^*CreERT2*^ mice. **a** *.* PHD1, 2, and 3 deletion efficiency in the gastrocnemius muscles of tamoxifen-treated *Phd2* ^*f/f*^ */Rosa26* ^*CreERT2*^ mice was determined using qRT-PCR. Relative gene expression was determined using gastrocnemius muscle tissue cDNA (*n* = 3–4 mice per group). **b** *.* Anti-PHD2 and PHD3 Western blotting of gastrocnemius and soleus muscles at 6 weeks after tamoxifen administration. **c** *.* The expression of HIF-1α in gastrocnemius and soleus at 5 weeks after tamoxifen administration. **d**. The level of hif-1α mRNA in gastrocnemius at 5 weeks after tamoxifen administration. *p < 0.05; **p < 0.01 compared to control. Values are means ± SEM

To inhibit the activation of PHD2 at the adult stage, we generated *Phd2* ^*f/f*^ */Rosa26* ^*CreERT2*^ mice. The administration of tamoxifen at 8–12 weeks of age activated Cre recombinase and resulted in the generation of cKO mice. We confirmed the deletion of *Phd*2 by examining protein levels from the *Phd2* ^*f/f*^ */Rosa26* ^*CreERT2*^and *Phd2* ^*f/f*^ mice after tamoxifen administration (Fig. 1b). In addition, we evaluated the efficiency of *Phd*2 deletion in the both gastrocnemius and soleus muscles of tamoxifen-treated *Phd2* ^*f/f*^ */Rosa26* ^*CreERT2*^ mice using quantitative reverse transcription polymerase chain reaction (qRT-PCR). The mRNA levels of *Phd2* were decreased by 46 % in the *Phd2* cKO mice compared with the *Phd2* ^*f/f*^ mice (Fig. 1a). On the contrary, mRNA levels of *Phd3* were significantly increased; however, the protein content of PHD3 was not changed in gastrocnemius muscle of PHD2-deficient mice (Fig. 1a, b). In addition to the mRNA, levels of *Phd1* were not changed by PHD2 deletion in gastrocnemius muscle (Fig. 1a).

### Changes in blood profile and muscle phenotype from PHD2 deletion

**Fig. 2:**
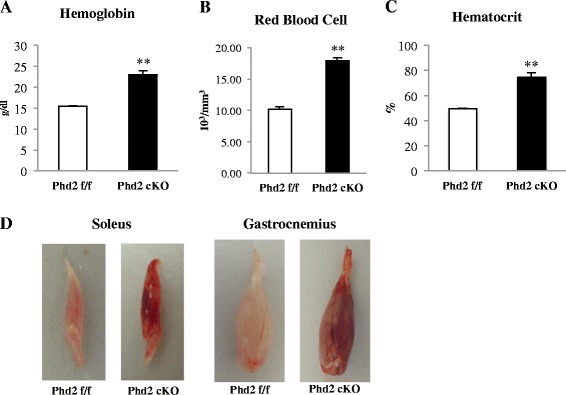
Changes in blood profile and the altered phenotype of skeletal muscle. Changes in blood profile at 6 weeks after tamoxifen administration. **a** Red blood cell count. **b** Hemoglobin level. **c** Hematocrit value (*n* = 10 per group). **d** Appearance of skeletal muscles, including the soleus and gastrocnemius, in Phd2^f/f^ and Phd2 cKO mice. **p < 0.01 compared to control. Values are means ± SEM

To monitor the change in blood profile in response to PHD2 inactivation, we obtained blood samples from the facial veins of both the *Phd2* ^*f/f*^ and *Phd2* ^*f/f*^ */Rosa26* ^*CreERT2*^ mice. Deleting *Phd2* caused changes to blood components: hemoglobin, red blood cells (RBCs), and hematocrit were all significantly increased in the *Phd2* cKO mice at 4 weeks after tamoxifen treatment (Fig. 2a-c). In addition, PHD2 deficiency led to changes in skeletal muscle phenotypes. Normally, the soleus is a slow fiber-enriched muscle, whereas the gastrocnemius is a fast fiber-enriched muscle. However, both the soleus and gastrocnemius muscles of the *Phd2* cKO mice showed a redder coloration than those of the *Phd2* ^*f/f*^ mice (Fig. 2d). Furthermore, the body weight and skeletal muscle weight (of both the soleus and gastrocnemius) were significantly decreased in the *Phd2* cKO mice compared with the Phd2^f/f^ mice (Table 1).

**Table 1:** Body weight and skeletal muscle mass

	*Phd2f/f*	*Phd2* cKO	*p* value
	*n* = 11	*n* = 13	
Soleus (mg)	8.57 ± 0.91	5.79 ± 1.3	<0.01
Gastrocnemius (mg)	161.85 ± 14.82	116.15 ± 18.46	<0.01
Soleus/BW (mg/g)	0.31 ± 0.03	0.26 ± 0.04	<0.01
Gastrocnemius/BW (mg/g)	5.92 ± 0.22	5.26 ± 0.25	<0.01
Body weight (g)	27.3 ± 2.42	22.0 ± 2.72	<0.01

Skeletal muscle and body weight were significantly decreased by PHD2 deficiency (*n* = 15 per group). *p < 0.05; **p < 0.01 compared to control. Values are means ± SEM

### *Phd2* deletion induces angiogenesis

**Fig. 3:**
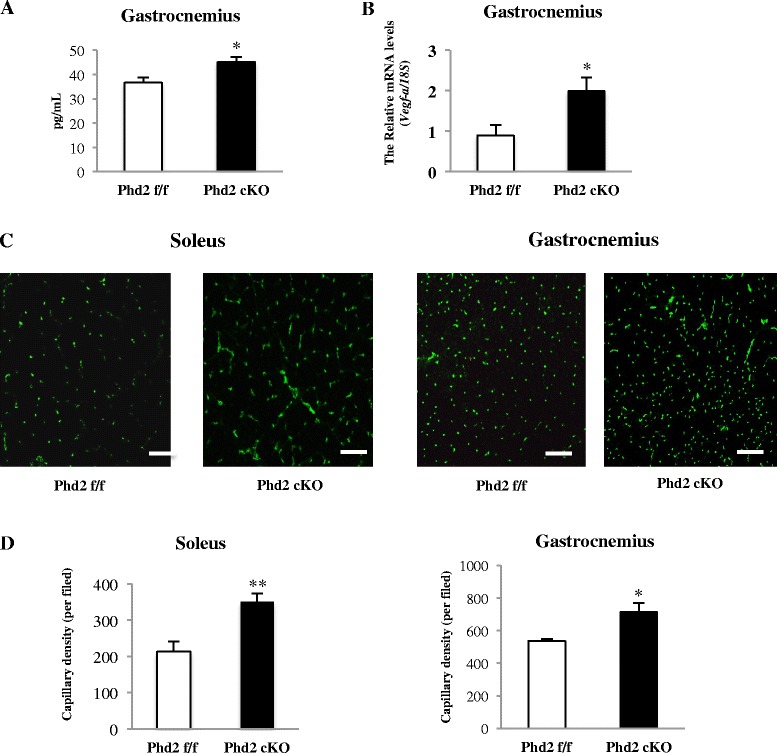
*Phd2* deletion induces angiogenesis in skeletal muscle. **a** The VEGF level in skeletal muscle was measured by ELISA (*n* = 3 per group). **b** Relative gene expression was measured using gastrocnemius muscle tissue cDNA (*n* = 3 per group). **c**, **d** The capillary density in skeletal muscles was determined by detecting CD31-positive cells using immunostaining (*n* = 5 per group). *p < 0.05, compared to control. Values are means ± SEM (*scale bar* = 100 μm)

To verify the hypoxic adaptation response resulting from *Phd2* deletion, we confirmed the expression of HIF-1α in both the gastrocnemius and soleus muscle in *Phd2* cKO mice (Fig. 1c). In contrast, the level of *hif-1α* mRNA was not changed in gastrocnemius (Fig. 1d). Additionally, we determined VEGF concentrations (Fig. 3a) and *Vegf-a* mRNA levels in gastrocnemius muscle (Fig. 3b) and monitored the capillary density in skeletal muscles by immunostaining for CD31, which is an endothelial cell marker in both the soleus and gastrocnemius. We were therefore able to confirm the expression of VEGF in the gastrocnemius, and we found that CD31-positive cells were significantly increased in both the soleus and gastrocnemius muscles of the *Phd2* cKO mice (Fig. 3c, d).

### The inactivation of PHD2 results in a skeletal muscle fiber-type transition toward slow fiber type

**Fig. 4:**
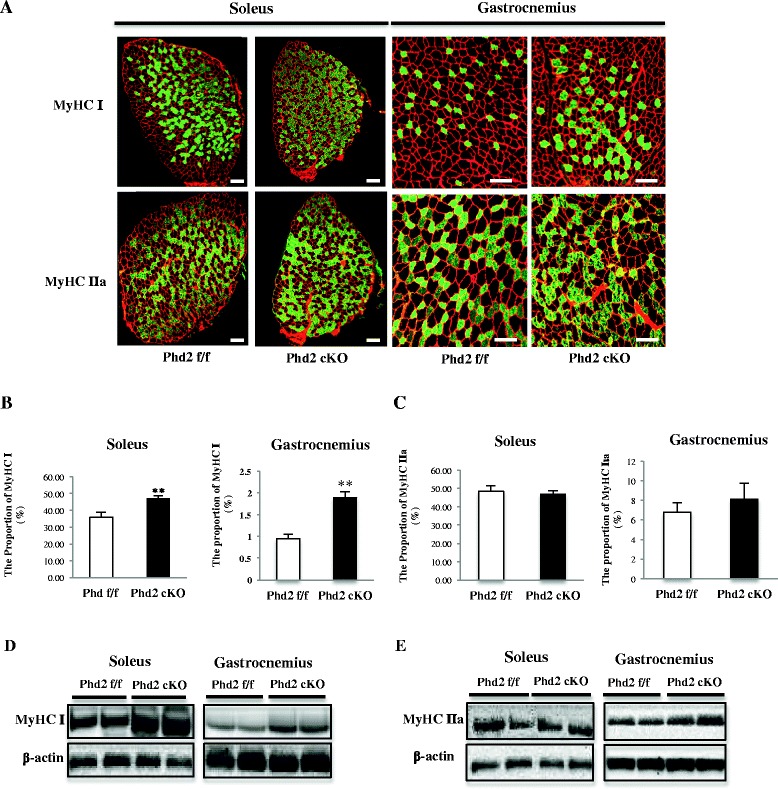
PHD2 deficiency induces slow muscle fiber type switching in the soleus and gastrocnemius muscles. **a** The composition of muscle fiber type was analyzed by immunostaining. Frozen sections of the soleus and gastrocnemius muscle at 6 weeks after tamoxifen treatment were stained with antibodies for MyHC I/slow (*green*) and MyHC IIa/fast (*green*) and counterstained for laminin (*red*). **b**-**c** The proportion of MyHC I and MyHC IIa muscle fibers was measured using ImageJ software (*n* = 5 per group). **d**-**e**Western blotting confirmed the MyHC I and MyHC IIa protein levels in the soleus and gastrocnemius muscles of Phd2^f/f^ and Phd2 cKO mice (*n* = 4 per group). **p < 0.01 compared to control. Values are means ± SEM (*scale bar* = 100 μm)

**Fig. 5:**
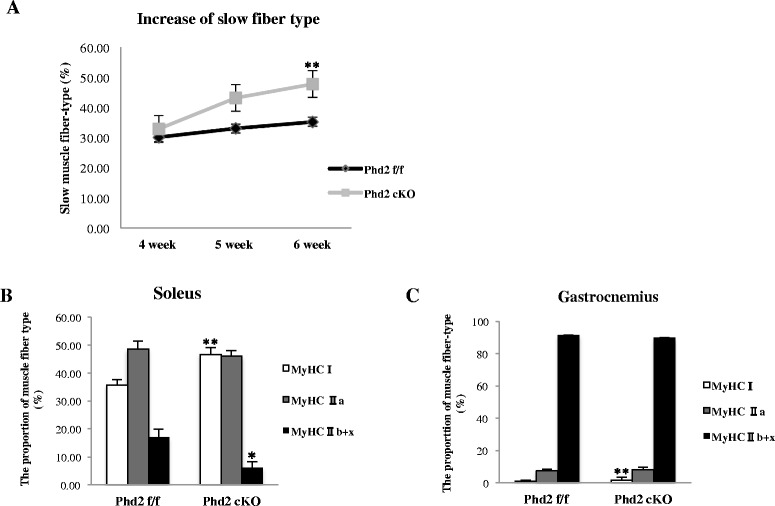
The time-dependent increase of the slow muscle fiber type and the distribution of muscle fiber types. **a** The increase in the slow muscle fiber type was analyzed in a time-dependent manner (*n* = 5 per group). **b**-**c**. The distribution of muscle fiber types in the soleus and gastrocnemius muscles at 6 weeks after tamoxifen administration (soleus *n* = 5, gastrocnemius *n* = 4 per group). *p < 0.05; **p < 0.01 compared to control. Values are means ± SEM

The inactivation of PHD2 in the whole body, including in skeletal muscles, resulted in muscle fiber-type conversion toward the slow fiber type. We demonstrated an increase in slow muscle fibers in the soleus and gastrocnemius muscles and used immunostaining to enumerate the number of muscle fibers. The proportion of slow muscle fibers in the *Phd2* cKO mice significantly increased in both the soleus muscle (35.8 % in *Phd2* ^*f/f*^ vs. 46.7 % in *Phd2* cKO, *p* < 0.01) and the gastrocnemius muscle (0.94 % in *Phd2* ^*f/f*^ vs. 1.89 % in *Phd2* cKO, *p* < 0.01) (Fig. 4b). In addition, the number of type IIa muscle fibers in the gastrocnemius was increased in the *Phd2* cKO mice (6.79 % in *Phd2* ^*f/f*^ vs. 9.27 % in *Phd2* cKO, n.s) (Fig. 5c) but was not significantly altered in the soleus muscle (48.48 % in *Phd2* ^*f/f*^ vs. 46.88 % in *Phd2* cKO, n.s) (Fig. 4c). Type IIx + b (unstained) fibers significantly decreased in the soleus muscles of the Phd2 cKO mice (16.9 % in *Phd2* ^*f/f*^ vs. 6.24 % in *Phd2* cKO) (Fig. 5b), whereas Type IIx + b fibers slightly decreased in the gastrocnemius of the Phd2 cKO mice (92.25 % in *Phd2* ^*f/f*^ vs. 89.9 % in *Phd2* cKO) (Fig. 5c). The muscle fiber-type conversion observed in the *Phd2* cKO mice indicated that the change in the proportion of slow fiber type was time-dependent. Slow muscle fibers increased from 5 and 6 weeks after tamoxifen treatment. However, at 4 weeks after tamoxifen, there was no difference in the proportion of muscle fiber types between the *Phd2* cKO and *Phd2* ^*f/f*^ mice (Fig. 5a). Additionally, Western blotting indicated an increase in MyHC I protein levels in both the soleus and the gastrocnemius (Fig. 4d), whereas MyHC IIa protein levels remained unchanged in both muscles (Fig. 4e). These data are in agreement with the histological results.

### A calcineurin/NFATc1-dependent pathway, but not the PGC-1α pathway, may be involved in the muscle fiber transition

**Fig. 6:**
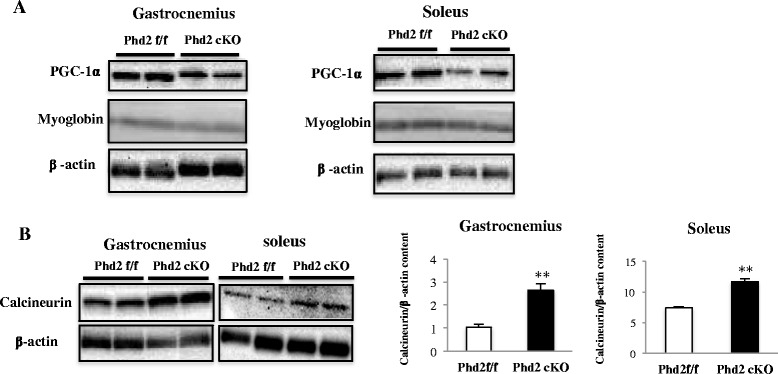
Expression of calcineurin, PGC-1α and myoglobin, and calcineurin in soleus and gastrocnemius muscles. **a** Western blotting analysis of protein lysates from the gastrocnemius and soleus for PGC-1α and myoglobin. **b** Calcineurin protein levels were measured using Western blotting in the gastrocnemius at 5 weeks after tamoxifen administration (*n* = 3 per group)

**Fig. 7:**
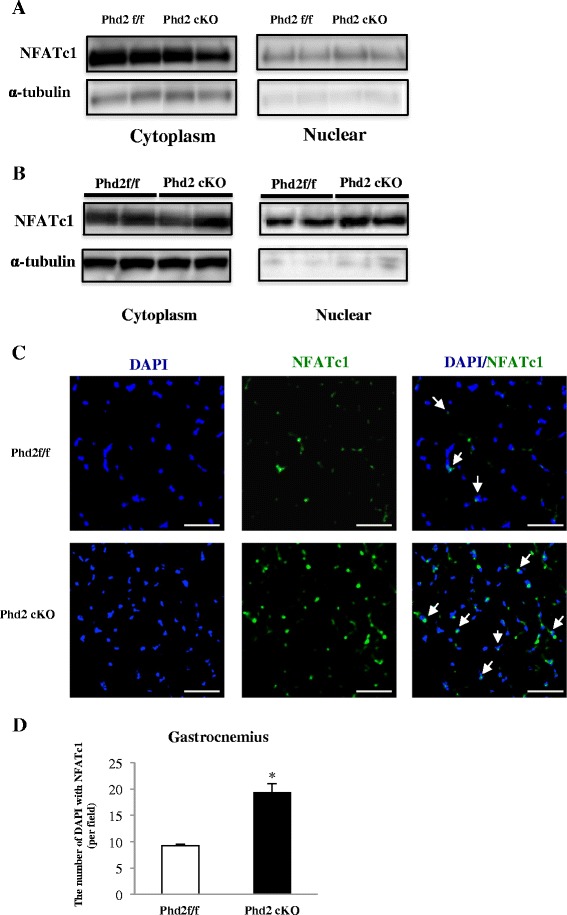
NFATc1 nuclear translocation in soleus and gastrocnemius muscles. **a**–**b** Western blotting analysis for the purity of cytoplasm/nuclear fractions in the soleus and gastrocnemius muscles at 4 weeks after tamoxifen administration (*n* = 3 per group). *p < 0.05; **p < 0.01 compared to control. Values are means ± SEM. **c** Immunostaining showed nuclei stained with NFATc1 in gastrocnemius. **d** The number of nuclei stained with NFATc1 (*n* = 4 per group). *p < 0.05, compared to control. Values are means ± SEM (magnification ×60, *scale bar* = 50 μm)

We demonstrated that deleting PHD2 promoted angiogenesis via HIF-1α stabilization and enhanced the slow muscle fiber type in both the soleus and gastrocnemius muscles. We examined several factors associated with muscle fiber transition, such as PGC-1α, myoglobin, calcineurin, and NFATc1, to investigate the mechanism underlying the induction of slow muscle fiber formation. The expression of PGC-1α decreased in the *Phd2* cKO mice while myoglobin expression was unchanged in both the soleus and gastrocnemius (Fig. 6a). In contrast, the expression of calcineurin was significantly increased in both muscles of *Phd2* cKO mice (Fig. 6b). Furthermore, we verified the nuclear translocation of NFATc1 in both muscles in the *Phd2* cKO mice using immunoblotting (Fig. 7a, b) and immunostaining (Fig. 7c, d).

### FK-506 suppresses slow fiber-type formation, but not capillary density in the soleus of *Phd2* cKO mice

**Fig. 8:**
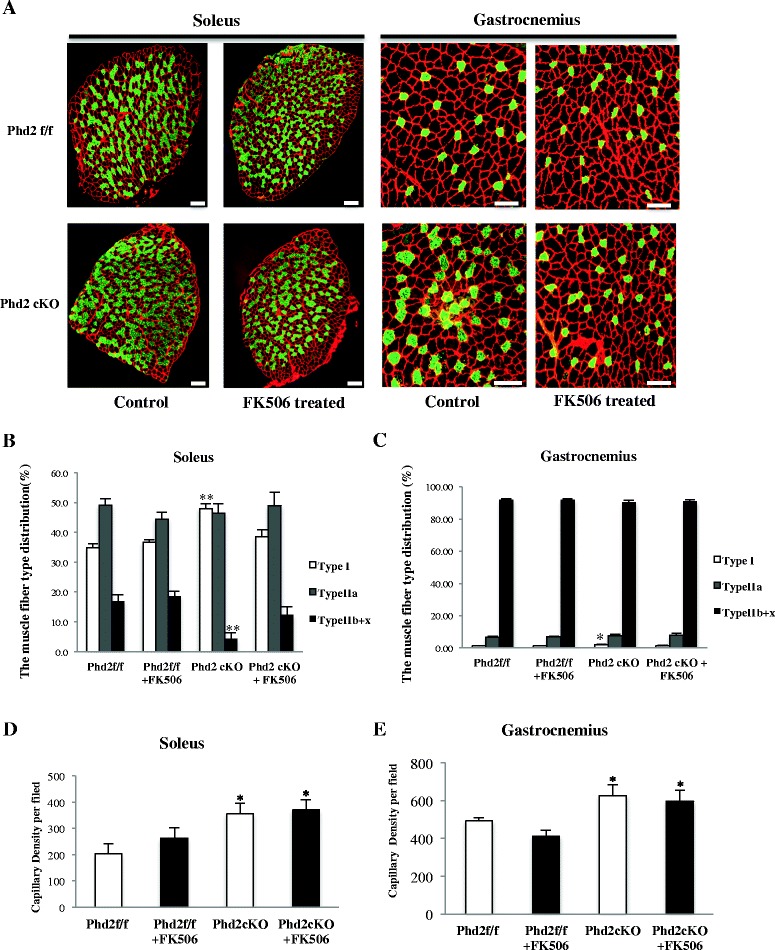
The effect of FK-506 treatment on the myosin heavy chain (MyHC) composition in soleus muscle of *Phd2-*deficient mice. **a** The composition of muscle fiber type was analyzed by immunostaining after the treatment of FK-506. Frozen sections of the soleus muscle taken at 6 weeks after tamoxifen treatment were stained with antibodies for myosin heavy chain (MyHC) I/slow (*green*) and counterstained for laminin (*red*) (*scale bar* = 100 μm). **b**-**c** The treatment of FK-506 for 7 days at 4 week after tamoxifen administration suppressed the increase in the proportion of slow muscle fiber type in both soleus and gastrocnemius muscles (*n* = 4 per group). **d**-**e** The capillary density in soleus and gastrocnemius muscles was determined by detecting CD31-positive cells after FK-506 treatment using immunostaining (*n* = 5 per group). *p < 0.05; **p < 0.01 compared to control. Values are means ± SEM

To verify the role of calcineurin/NFATc1 signaling in slow muscle fiber-type transition, we administrated FK-506, which is a calcineurin inhibitor, at 4 weeks after tamoxifen treatment for seven consecutive days. The administration of FK-506 suppressed slow muscle fiber-type transition in both soleus (35.5 % in *Phd2* ^*f/f*^ vs. 36.8 % in *Phd2* ^*f/f*^ + FK-506, 48 % in *Phd2* cKO vs. 38.6 % in *Phd2* cKO + FK-506, *p* < 0.01) and gastrocnemius (1.17 % in *Phd2* ^*f/f*^ vs. 1.13 % in *Phd2* ^*f/f*^ + FK-506, 2.03 % in *Phd2* cKO vs. 1.29 % in *Phd2* cKO + FK-506, *p* < 0.01) of Phd2 cKO mice (Fig. 8a–c). PhD cKO mice had larger capillary density as compared to control mice. FK-506 treatment, however, did not affect the capillary density (Fig. 8d, e).

### Chronic hypoxic exposure induces the alteration of blood components and skeletal muscle phenotype

Ten percent of hypoxic exposure for 4 weeks induced the dramatic elevation of red blood cells, hemoglobin, and hematocrit (Additional file 1: Figure S5A–C). Also, the mice exposure to hypoxic condition showed the significant decrease of body weight. By contrast, there was no change in the muscle mass of the soleus and gastrocnemius muscles (Additional file 1: Figure S5D). Additionally, both the soleus and gastrocnemius muscles of the hypoxic mice showed a redder coloration than those of the normoxic mice (Additional file 1: Figure S5E). Ten percent of hypoxic exposure for 4 weeks showed the increase of type IIa in both soleus (47.84 % in normoxia vs. 47.74 % in normoxia + FK-506, 49.72 % in hypoxia + FK-506 vs. 55.05 % in hypoxia, n.s) and gastrocnemius (6.76 % in normoxia vs. 6.82 % in normoxia + FK-506 vs. 11.24 % in hypoxia, *p* < 0.05) muscle, but not type I. Moreover, type IIb + x fibers were significantly reduced by hypoxic exposure in both the soleus (17.89 % in normoxia, 15.41 % in normoxia + FK-506, and 15.39 % in hypoxia + FK-506 vs. 6.56 % in hypoxia *p* < 0.05) and gastrocnemius (92.18 % in normoxia vs. 92.05 % in normoxia + FK-506 vs. 87.19 % in hypoxia, *p* < 0.05). Additionally, the suppression of calcineurin activity by FK-506 prevented the decrease of type IIb + x in soleus, whereas FK-506 treatment did not show the significant differences in gastrocnemius muscle compared with hypoxia mice (Additional file 1: Figure S5F).

## Discussion

There are several reports that the expression of HIF-1α by hypoxia may link to the expression of slow muscle fiber phenotype. For instance, the exposure to 8 % of hypoxic and normobaric condition showed the high proportion of oxydative phenotype in mice and the suppression of oxidative metabolism in skeletal muscle [12]. Furthermore, they provided information that HIF-1α siRNA treatment suppressed the increase of MyHC I mRNA in cultured C2C12 myotubes compared with control under 4 % hypoxic condition. [13]. Even though HIF-1α seems to be one of the crucial regulators of muscle fiber-type transition toward slow fiber type, the downstream of the muscle fiber-type transition under HIF-1α-dependent pathway remains unanswered. Here, we demonstrated that HIF-1α stabilization by PHD2 knockout elicited a transition of skeletal muscle fiber type toward the slow fiber type and that this may have occurred through the activation of calcineurin/NFATc1 signaling pathway in vivo*.*

PHD2 is one of the oxygen-level-dependent PHD isoforms that regulate the stabilization of HIF-α under hypoxic conditions [16, 18]. We confirmed that the deletion of PHD2 resulted in the stabilization of HIF-α in skeletal muscles. The inactivation of prolyl hydroxylase is known to lead to HIF-α stabilization and translocation into the nucleus, where it regulates a variety of target genes related to angiogenesis, erythropoiesis, and energy metabolism [26]. In addition, while we did not find any change in PHD1 mRNA, we found an increase in PHD3 mRNA level in gastrocnemius in PHD2 deficient mice. However, PHD3 protein content was not altered in gastrocnemius muscle of PHD2 deficient mice. This expression profile of PHD mRNAs may be as a result of increase in HIF-1α, because PHD2 and PHD3 genes contain hypoxia response element (HRE) and consequently are transcriptionally up-regulated by HIF-1α, which in return may rapidly remove HIF-1α as a negative feedback [27, 28]. The reason why PHD3 protein was not altered remains unknown. It is possible that despite of PHD2 deficiency, PHD3 may partly substitute hydroxylation of HIF-1α. It is apparent; however, the activity of PHD3, if any, was insufficient to abrogate HIF-1α in PHD2 deficient mice.

The stabilization of HIF-1α by PHD2 deficiency has been shown to activate erythropoietic and angiogenic responses [22, 29, 30]. Indeed, we confirmed dramatic elevations in RBCs, hemoglobin, and hematocrit in blood samples in the *Phd2* cKO mice in this study. Interestingly, myoglobin levels did not change in either the soleus or the gastrocnemius muscle in this study. Therefore, the reddish appearance of skeletal muscle tissue may be attributed to the marked increased in the red blood cell in the blood stream. These data are consistent with a previous report that hypoxia-mediated transcriptional activation of myoglobin gene expression was independent of HIF-1 [31]. Additionally, PHD2-deficient mice showed the loss of muscle mass, the decreased muscle fiber size, and the reduced food intake (Additional file 2: Figure SA, B). A previous study demonstrated that 8 % of hypoxic condition led to muscle atrophy via proteasome ubiquitin system compared to control and pair-fed mice [32]. Possible causes of loss of muscle mass and reduced muscle fiber size in our study may be because of the significant reduction in food intake of PHD2-deficient mice. In addition, we also detected a significant increase in the mRNA level of atrogin-1, a potent ubiquitin E3 ligase, in gastrocnemius muscle in PHD2-deficient mice (Additional file 3: Figure S4A). These results indicate possibility that the stabilization of HIF-1α may involve in the type II fibers atrophy via both decreased food intake and the proteasomal processing of skeletal muscle specific proteins.

Previous work has shown that erythropoietin contributed to slow muscle fiber-type gene expression via PGC-1α, a master regulator of mitochondrial biogenesis [33, 34]. Our study, however, demonstrated that PGC-1α expression was negatively regulated by PHD2 deficiency in both the soleus and gastrocnemius muscles despite the activation of erythropoiesis. Additionally, mtDNA ratio, carnitine acyltransferase1b (CPT1b), and acyl-CoA dehydrogenase, long chain (ACADL) were not changed by PHD2 deficiency in skeletal muscle (Additional file 4: Figure S1). In an additional experiment, PGC-1α was not changed by PHD2 knockdown in cultured C2C12 myotubes (Additional file 5: Figure S3B), which agrees with the previous report that PGC-1α was suppressed under hypoxic conditions in C2C12 myotubes in vitro [13]. Furthermore, we detected the increase of SIRT1 mRNA level in gastrocnemius muscle of PHD2-deficient mice (Additional file 4: Figure S1C). Although we did not directly measure acetylation of PGC-1α, it is possible that SIRT1 pathway may have been involved in the suppression of PGC-1α activity through acetylation in vivo. Thus, skeletal muscle fiber-type transition toward the slow fiber type seems to be independent of PGC-1α in the PHD2-deficient mice.

Calcineurin, which is activated by the increase of intracellular calcium [35], is well known as a pivotal regulator of the oxidative muscle phenotype [36]. Skeletal muscle-specific transgenic mice expressing calcineurin showed endurance exercise capacity [37]. A recent study reported that an increase in steady-state cytoplasmic and intracellular Ca^2+^ was observed under hypoxia in skeletal muscle tissue [31]. Furthermore, increased intracellular Ca^2+^ under hypoxia was shown to lead to the activation of a calcineurin/NFAT pathway in skeletal muscle [31, 38]. The activation of calcineurin results in the dephosphorylation of NFATs, the main downstream targets of calcineurin, leading to their nuclear translocation and the triggering of transcription factors related to slow fiber-type formation [39]. PHD2-deficient mice in this study clearly demonstrated NFATc1 nuclear translocation in both gastrocnemius and soleus muscles. Each NFAT isoform (c1, c2, c3, and c4) not only plays a role in the development and growth of skeletal muscle but also regulates the transition toward slow muscle fiber type [39, 40]. In particular, NFATc1 interacts with MyoD, myocyte enhancer factor 2 (MEF2), and the transcriptional coactivator p300 [36, 39, 41] and enhances the expression of MyHC I in rodent skeletal muscle [5]. Indeed, we monitored the alteration of NFATc1 and MEF2, which are known as a downstream factor of calcineurin, in cultured C2C12 myotubes after PHD2 siRNA treatment. PHD2 knockdown induced the expression of NFATc1 and the increase of slow myosin heavy chain, whereas the amount of MEF2 protein was not significantly changed in cultured myotubes (Additional file 5: Figure S3B). Because multiple factors maybe involved in fiber-type transition, although MEF2 was not actively regulated under PHD2 deficiency, it does not rule out the involvement of MEF2 in the fiber-type transition under PHD2 deficiency.

Because the treatment of calcineurin inhibitor tacrolimus (FK-506) is known to prevent overload-induced fiber-type transition in mice skeletal muscle from IIb, IIx, and IIa, to I through the dephosphorylation of NFATc1 [42], we administered FK-506 to verify that calcineurin was responsible for the skeletal muscle fiber-type transition in the PHD2-deficient mice. It successfully suppressed slow muscle fiber-type transition suggesting that calcineurin is one of the key regulators for muscle fiber-type transition toward the slow fiber-type under the activation of a hypoxic-responsive pathway in PHD2-deficient mice.

In order to confirm the validity of the PHD2 deficiency as a model of hypoxic adaptation of skeletal muscle, we exposed wild-type mice to actual hypoxia of 10 % oxygen for 4 weeks and examined the distribution of fiber types in soleus and gastrocnemius muscles. Although the hematological changes under real hypoxia was milder as compared to PHD2 deficiency, there was significant changes in the fiber-type distribution in soleus and gastrocnemius muscles under real hypoxia. Because the changes in fiber-type distribution were effectively abrogated under FK-506, we consider calcineurin pathway was responsible for fiber-type transition under real hypoxia as well as under PHD2 deficiency. The reason why type IIa instead of type I was increased in gastrocnemius is unknown, but it is possible that the effect of real hypoxia was somewhat milder as compared to PHD2 deficiency.

Overall, we demonstrated that the hypoxic response by HIF-α stabilization in PHD2-deficient mice was linked to the alteration of skeletal muscle phenotype via a calcineurin/NFATc1 signaling pathway. Although the mechanism by which the HIF-α stabilization resulted in the activation of calcineurin/NFATc1 signaling is yet to be elucidated, there are several possibilities for how this activation may take place in muscle tissue. Previous work has reported that hypoxia resulted in the up-regulation of VEGF as well as the elevation of calcium in skeletal muscle tissue [10, 31]. In endothelial cells, VEGF is known to be expressed by HIF-α, which binds to the hypoxia-responsive element (HRE) in the VEGF-A promoter [43]. VEGF principally binds to VEGFRs on the endothelial cell surface [43]. Ligand-bound VEGFRs provoke intracellular Ca^2+^ mobilization through nicotinic acid adenine dinucleotide phosphate (NAADP) in vascular endothelial cells [44, 45]. In these cells, the elevation of intracellular Ca^2+^ level induced by VEGF and VEGFR leads to the activation of calcineurin and the nuclear translocation of NFATc1 [46]. Although we lack information regarding the competent expression of VEGFR on skeletal muscle cells, the administration of recombinant VEGF to cultured C2C12 myotubes is known to increase troponin I mRNA expression, which is another hallmark of type I fibers [47]. Taken together, VEGF production from endothelial cells in the skeletal muscle tissue under a hypoxic response induced by PHD2 deficiency may lead to the activation of a calcineurin/NFATc1 signaling pathway resulting in the expression of MyHC I in skeletal muscle tissue. In the gastrocnemius muscle of PHD2-deficient mice, fiber-type transition to type I was limited only to the central part of the cross section of the muscle tissue. Since the increase in the capillaries under hypoxia was localized centrally, corresponding to the site of fiber-type transition, we suppose that endothelial cells may have been responsible for VEGF production, which then locally induced slow fiber-type transition. This idea seems to be concordant with the finding that suggests a pivotal role of the angiogenic response in the early stage of skeletal muscle fiber-type transition after voluntary running [48].

## Conclusions

In summary, the present study demonstrated that PHD2 conditional knockout mice may serve as a model for chronic HIF-1α stabilization as in mice exposed to low oxygen. Stabilized HIF-1α induced by PHD2 conditional knockout contributed to the transition of muscle fibers toward a slow fiber type via calcineurin/NFATc1 signaling pathway. Consequently, our findings may provide a basis for explaining the alteration of skeletal muscle phenotype in the HIF-α-stabilization.

## Additional files

Additional file 1: Figure S5. Chronic hypoxic exposure induces the alteration of blood components and skeletal muscle phenotype. Change in blood profile after exposure to 10 % hypoxic condition for 4 weeks. A*.* Red blood cell count. B*.* Hemoglobin level. C*.* Hematocrit value (*n* = 5–6 per group). D. Mass of soleus, and gastrocnemius of male under normoxia (Nx), normoxia treated with FK-506, hypoxia (Hx), and hypoxia treated with FK-506 at 8–10 weeks of age normalized to body weight (*n* = 5–6 mice per group). E. Appearance of skeletal muscles, including the soleus and gastrocnemius, in normoxia and hypoxia. F. The composition of muscle fiber-type was analyzed by immunostaining. Frozen sections of the soleus and gastrocnemius muscle after exposure to normoxia and hypoxia were stained with antibodies for MyHC I/slow, MyHC IIa and counterstained for laminin (*n* = 4–6 mice per group). *p < 0.05, **p < 0.01 compared to control. Values are means ± SEM. (PDF 5163 kb)

Additional file 2: Figure S2. Gastrocnemius muscle cross-sectional area (CSA), and food intake in PHD2^f/f^ and PHD2 cKO mice. A. Gastrocnemius muscle cross-sectional area of fast fiber at 6 weeks after tamoxifen administration. (*n* = 4 per group) B. Daily food intake in PHD2^f/f^ and PHD2 cKO mice (*n* = 2 per group). *p < 0.05, compared to control. Values are means ± SEM. (PDF 97 kb)

Additional file 3: Figure S4. The expression of genes related to ubiquitin proteasome system in skeletal muscle. The mRNA level of Murf-1 and antrogin-1 in gastrocnemius muscle (*n* = 4 per group). Values are means ± SEM. (PDF 58 kb)

Additional file 4: Figure S1. Mitochondrial biogenesis and oxidative metabolism was not changed by PHD2 deficiency. A. mitochondrial DNA content (*cytochome b* gene) in soleus and gastrocnemius muscles (*n* = 4 per group). B. The mRNA level of CPT1b and Acadl in soleus muscle (*n* = 4 per group). C. The mRNA level of SIRT1 in gastrocnemius muscle (*n* = 4 per group). Values are means ± SEM. (PDF 6007 kb)

Additional file 5: Figure S3 **PHD2 knockdown by siRNA transfection induced the increase of slow myosin heavy chain.** A. Experiment schematic for PHD2 siRNA transfection in cultured C2C12 myotubes. B. Immunoblotting revealed increase of HIF-1α, slow myosin heavy chain, NFATc1, and the suppression of PHD2 at 3 day after siPHD2 treatment. (PDF 769 kb)

## Abbreviations

cKO: conditional knock out
FK-506: tacrolimus
GAPDH: glyceraldehyde 3-phosphate dehydrogenase
HIF-α: hypoxia-inducible factor-α
Hx: hypoxia
mRNA: messenger RNA
MyHC: myosin heavy chain
NAADP: nicotinic acid adenine dinucleotide phosphate
NFAT: nuclear factor of activated T-cells
Nx: normoxia
PBS: phosphate-buffered saline
PFA: paraformaldehyde
PGC-1α: peroxisome proliferator-activated receptor gamma coactivator-1α
PHD2^f/f^: prolyl hydroxylase domain 2 flox/flox
VEGF: vascular endothelial growth factor
