# Supplementary figures and images for: Prolyl hydroxylase domain 2 deficiency promotes skeletal muscle fiber-type transition via a calcineurin/NFATc1-dependent pathway
Source: Skelet Muscle. 2016 Mar 5;6:5. doi: 10.1186/s13395-016-0079-5 (PMC4779261; doi:10.1186/s13395-016-0079-5)

Supplementary Figure 5

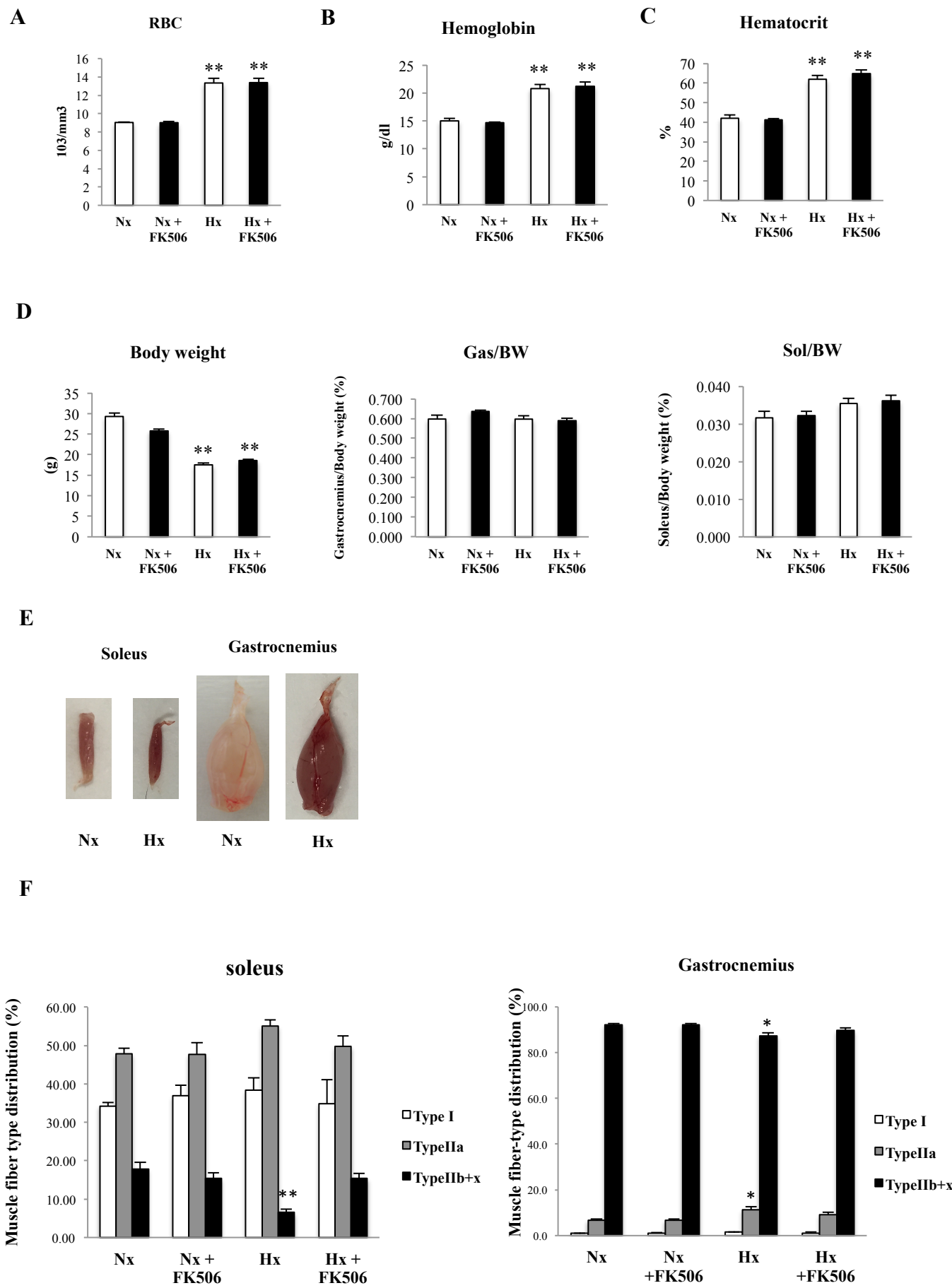

Supplement: Supplementary file 1 — Chronic hypoxic exposure induces the alteration of blood components and skeletal muscle phenotype. Change in blood profile after exposure to 10 % hypoxic condition for 4 weeks. A. Red blood cell count. B. Hemoglobin level. C. Hematocrit value (n = 5–6 per group). D. Mass of soleus, and gastrocnemius of male under normoxia (Nx), normoxia treated with FK-506, hypoxia (Hx), and hypoxia treated with FK-506 at 8–10 weeks of age normalized to body weight (n = 5–6 mice per group). E. Appearance of skeletal muscles, including the soleus and gastrocnemius, in normoxia and hypoxia. F. The composition of muscle fiber-type was analyzed by immunostaining. Frozen sections of the soleus and gastrocnemius muscle after exposure to normoxia and hypoxia were stained with antibodies for MyHC I/slow, MyHC IIa and counterstained for laminin (n = 4–6 mice per group). *p < 0.05, **p < 0.01 compared to control. Values are means ± SEM. (PDF 5163 kb) [file 13395_2016_79_MOESM1_ESM.pdf]

# Supplementary Figure 2

A

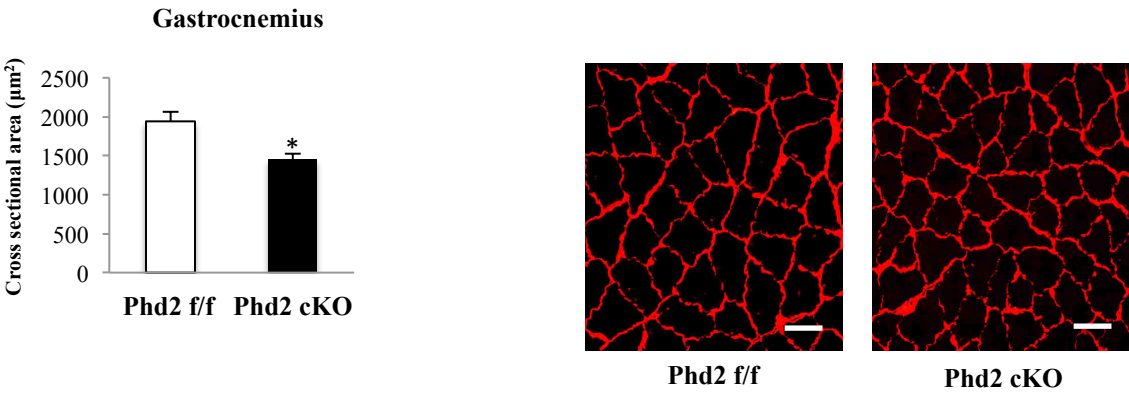

B

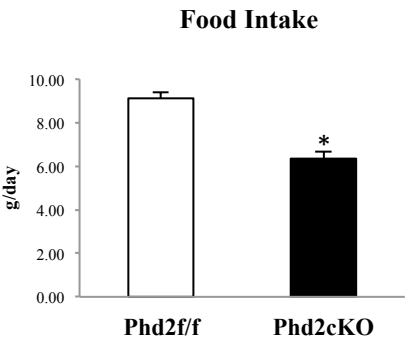

Supplement: Supplementary file 2 — Gastrocnemius muscle cross-sectional area (CSA), and food intake in PHD2f/f and PHD2 cKO mice. A. Gastrocnemius muscle cross-sectional area of fast fiber at 6 weeks after tamoxifen administration. (n = 4 per group) B. Daily food intake in PHD2f/f and PHD2 cKO mice (n = 2 per group). *p < 0.05, compared to control. Values are means ± SEM. (PDF 97 kb) [file 13395_2016_79_MOESM2_ESM.pdf]

# Supplementary Figure 4

A

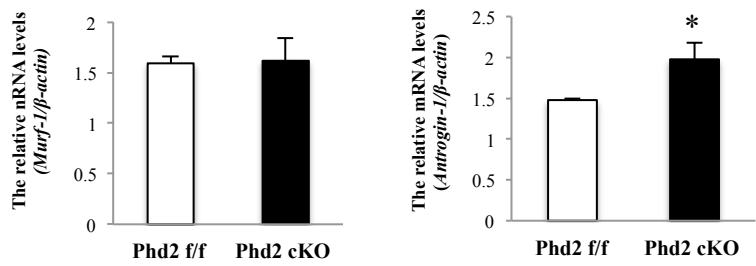

Supplement: Supplementary file 3 — The expression of genes related to ubiquitin proteasome system in skeletal muscle. The mRNA level of Murf-1 and antrogin-1 in gastrocnemius muscle (n = 4 per group). Values are means ± SEM. (PDF 58 kb) [file 13395_2016_79_MOESM3_ESM.pdf]

# Supplementary Figure 3

A

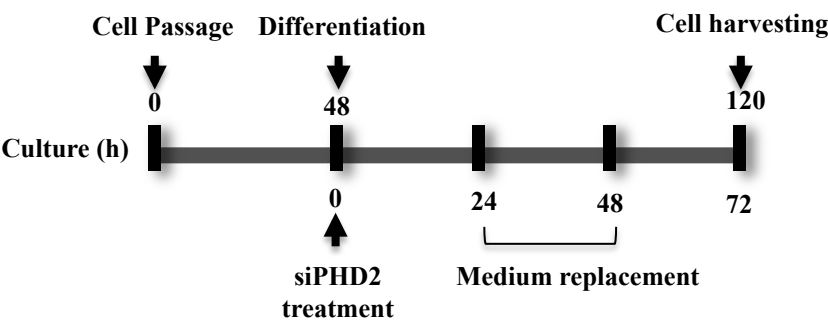

B

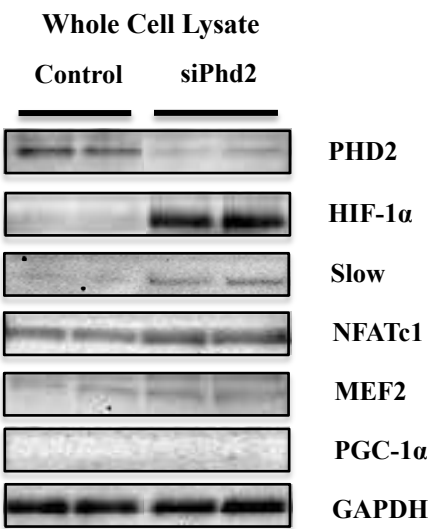

Supplement: Supplementary file 5 — PHD2 knockdown by siRNA transfection induced the increase of slow myosin heavy chain. A. Experiment schematic for PHD2 siRNA transfection in cultured C2C12 myotubes. B. Immunoblotting revealed increase of HIF-1α, slow myosin heavy chain, NFATc1, and the suppression of PHD2 at 3 day after siPHD2 treatment. (PDF 769 kb) [file 13395_2016_79_MOESM5_ESM.pdf]
