# Supplementary material for: Prolyl hydroxylase domain 2 deficiency promotes skeletal muscle fiber-type transition via a calcineurin/NFATc1-dependent pathway
Source: Skelet Muscle. 2016 Mar 5;6:5. doi: 10.1186/s13395-016-0079-5 (PMC4779261; doi:10.1186/s13395-016-0079-5)

Supplementary Figure 1

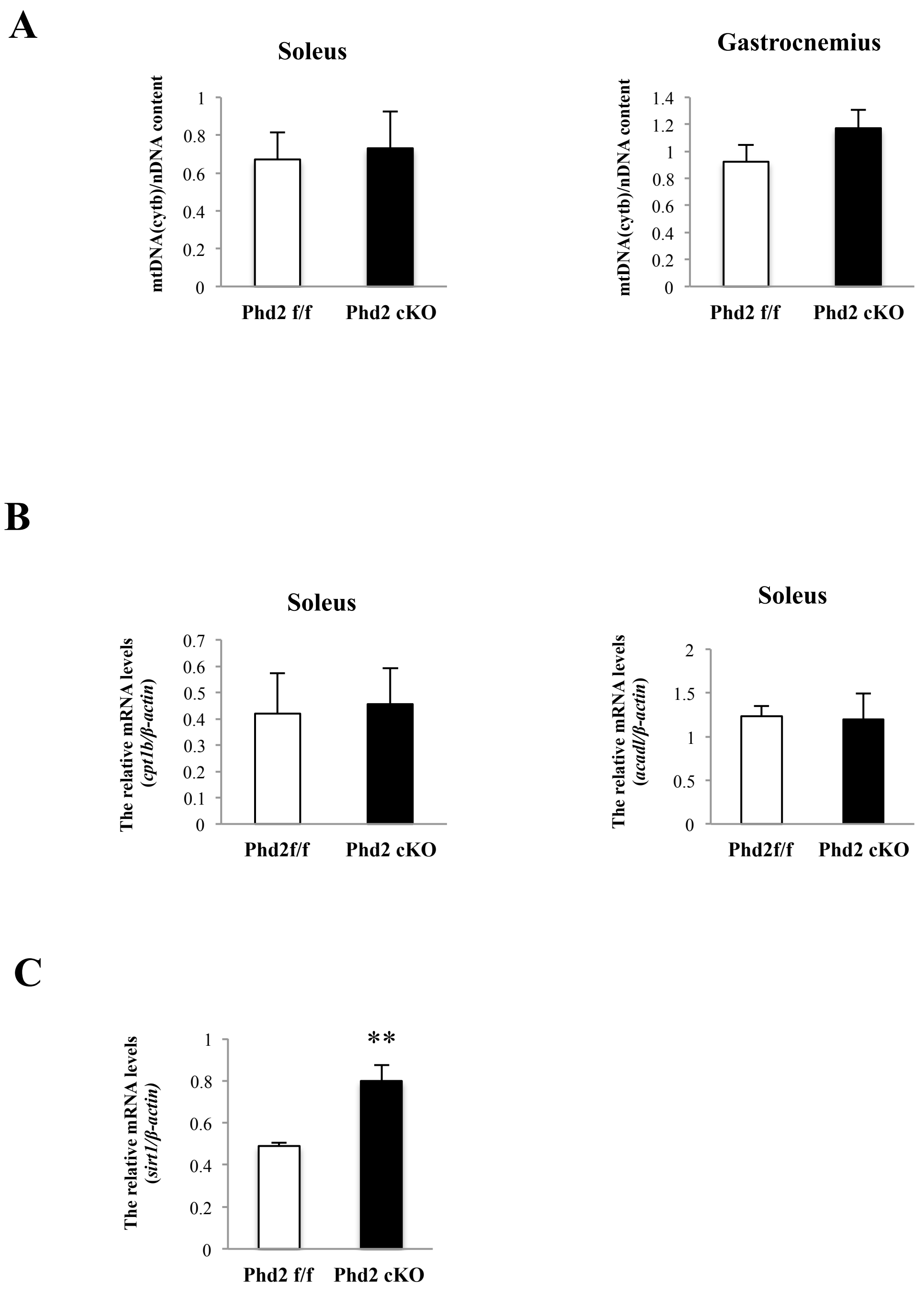

Supplementary Figure 2

A

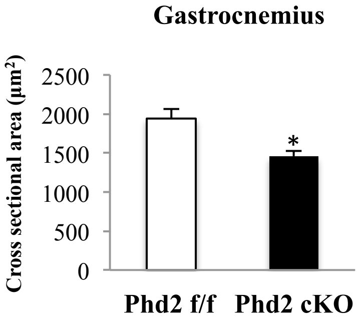

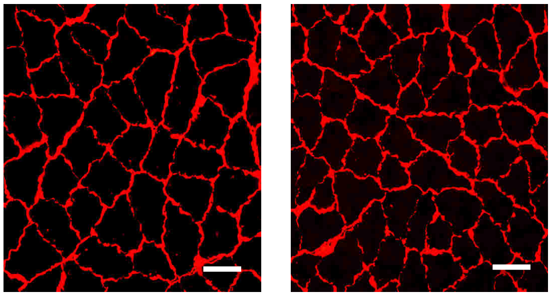

Phd2 f/f Phd2 cKO

B

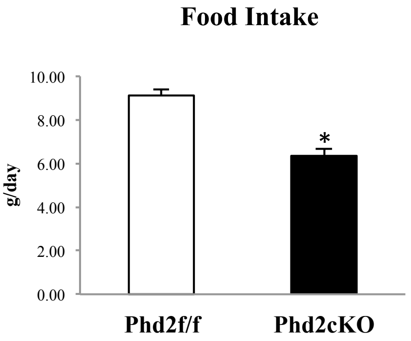

Supplementary Figure 3

A

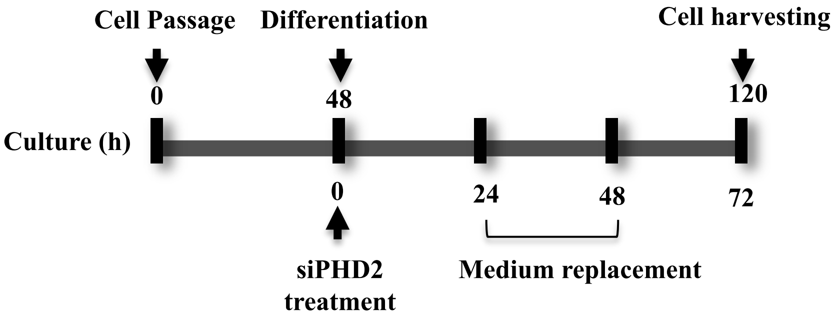

B

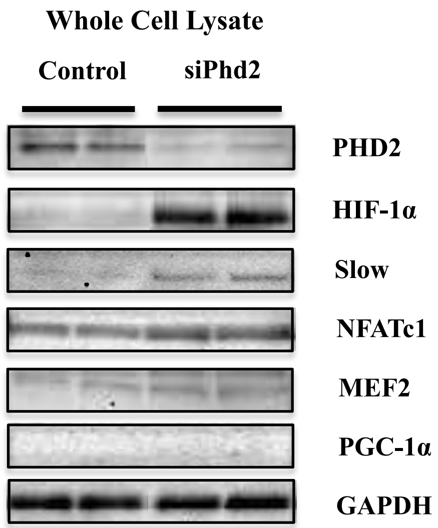

Supplementary Figure 4

A

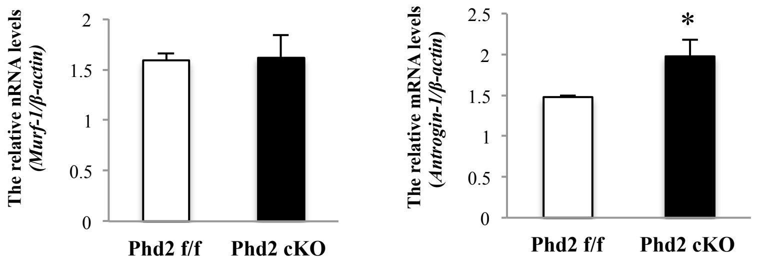

Supplementary Figure 5

A

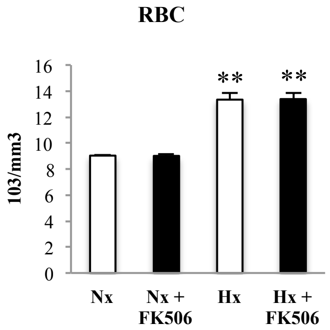

B

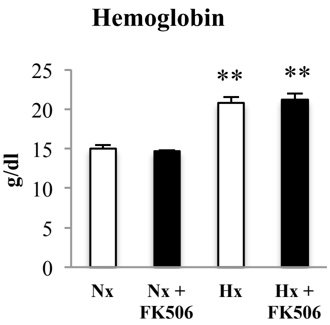

C

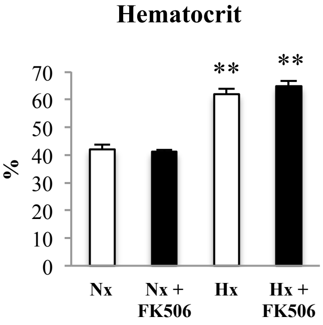

D

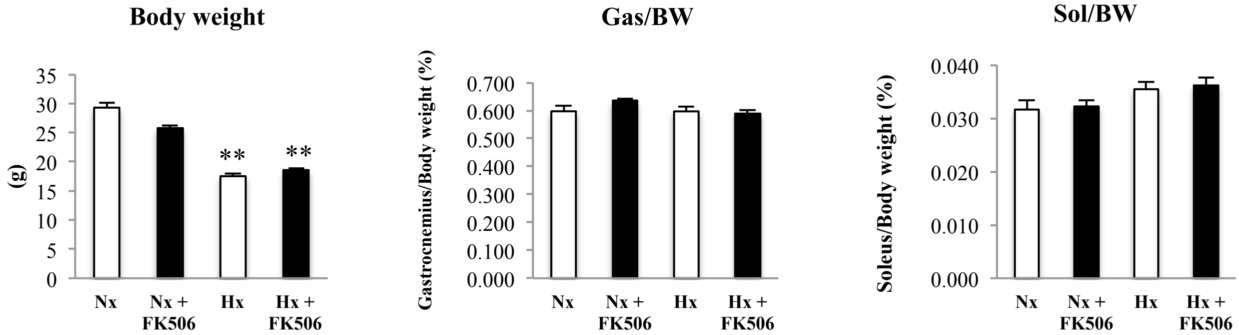

E

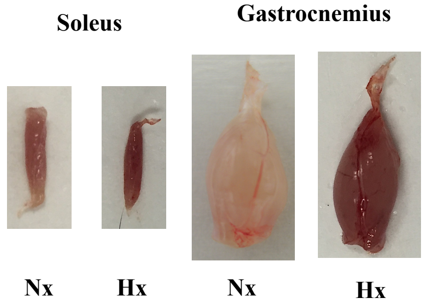

F

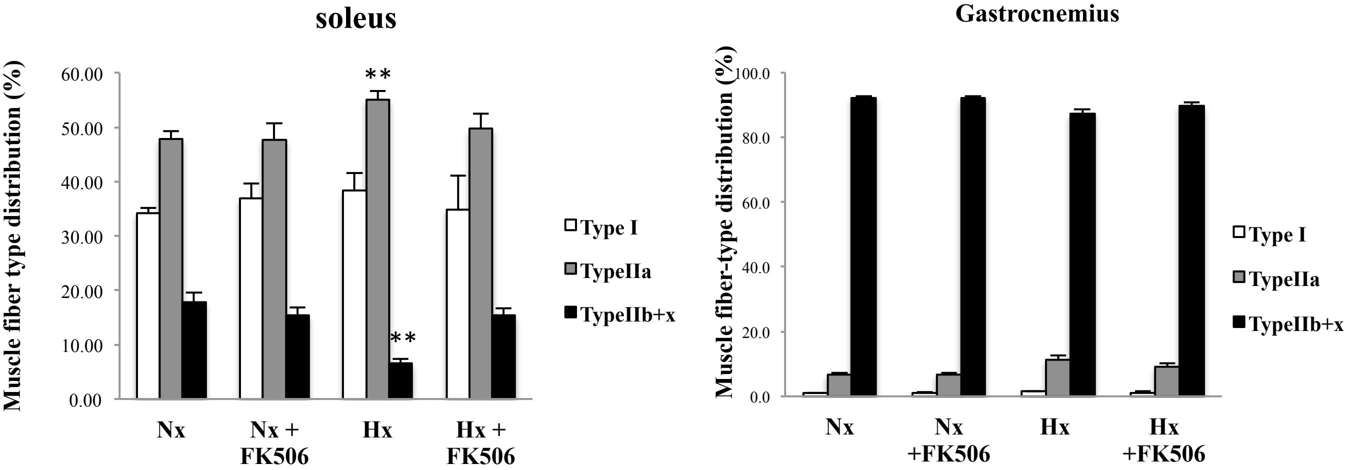

Supplement: Supplementary file 4 — Mitochondrial biogenesis and oxidative metabolism was not changed by PHD2 deficiency. A. mitochondrial DNA content (cytochome b gene) in soleus and gastrocnemius muscles (n = 4 per group). B. The mRNA level of CPT1b and Acadl in soleus muscle (n = 4 per group). C. The mRNA level of SIRT1 in gastrocnemius muscle (n = 4 per group). Values are means ± SEM. (PDF 6007 kb) [file 13395_2016_79_MOESM4_ESM.pdf]
